# The Information Needs and Experiences of People Living With Cardiac Implantable Electronic Devices: Qualitative Content Analysis of Reddit Posts

**DOI:** 10.2196/46296

**Published:** 2023-11-01

**Authors:** Mitchell Nicmanis, Anna Chur-Hansen, Karen Linehan

**Affiliations:** 1 School of Psychology Faculty of Health and Medical Sciences University of Adelaide Adelaide Australia

**Keywords:** implantable cardioverter defibrillator, pacemaker, cardiac resynchronization therapy, social media, patients, peer support, content analysis, experiences

## Abstract

**Background:**

Cardiac implantable electronic devices (CIEDs) are used to treat a range of cardiovascular diseases and can lead to substantial clinical improvements. However, studies evaluating patients’ experiences of living with these devices are sparse and have focused mainly on implantable cardioverter defibrillators. In addition, there has been limited evaluation of how people living with a CIED use social media to gain insight into their condition.

**Objective:**

This study aims to analyze posts from web-based communities called subreddits on the website Reddit, intended for people living with a CIED, to characterize the informational needs and experiences of patients.

**Methods:**

Reddit was systematically searched for appropriate subreddits, and we found 1 subreddit that could be included in the analysis. A Python-based web scraping script using the Reddit application programming interface was used to extract posts from this subreddit. Each post was individually screened for relevancy, and a register of participants’ demographic information was created. Conventional qualitative content analysis was used to inductively classify the qualitative data collected into codes, subcategories, and overarching categories.

**Results:**

Of the 484 posts collected using the script, 186 were excluded, resulting in 298 posts from 196 participants being included in the analysis. The median age of the participants who reported this was 33 (IQR 22.0-39.5; range 17-72) years, and the majority had a permanent pacemaker. The content analysis yielded 5 overarching categories: use of the subreddit by participants, questions and experiences related to the daily challenges of living with a CIED, physical sequelae of CIED implantation, psychological experiences of living with a CIED, and questions and experiences related to health care while living with a CIED. These categories provided insight into the diverse experiences and informational needs of participants living with a CIED. The data predominantly represented the experiences of younger and more physically active participants.

**Conclusions:**

Social media provides a platform through which people living with a CIED can share information and provide support to their peers. Participants generally sought information about the experiences of others living with a CIED. This was often done to help overcome a range of challenges faced by participants, including the need to adapt to living with a CIED, difficulties with navigating health care, psychological difficulties, and various aversive physical sequelae. These challenges may be particularly difficult for younger and physically active people. Health care professionals may leverage peer support and other aid to help people overcome the challenges they face while living with a CIED.

## Introduction

### Background

Cardiovascular diseases present a global health burden, with conditions such as atrial fibrillation being experienced by 37 million people globally [1] and heart failure experienced by 60 million people globally [2]. Cardiac implantable electronic devices (CIEDs) represent a growing treatment option for these conditions and a range of other cardiovascular diseases [3,4]. There are 3 common types of CIEDs: permanent pacemakers (PPMs), implantable cardioverter defibrillators (ICDs), and cardiac resynchronization therapy devices. In addition, novel CIEDs including subcutaneous ICDs (S-ICDs), and leadless PPMs have been developed (refer to the study by Steffen et al [4] for a detailed review). Qualitative studies have been particularly useful for elucidating the experiences of patients living with a CIED; however, the current literature has disproportionately focused on the experiences of people living with an ICD.

Systematic reviews of qualitative studies indicate that living with an ICD presents a number of psychological and physical challenges for patients owing to both the physical form and functions of these devices [5,6]. This includes experiences of anxiety owing to the defibrillatory shocks administered by a patient’s ICD, a well-documented phenomenon [7]. In addition, qualitative studies have evaluated decisions regarding the discontinuation of ICD-administered therapies during end-of-life care [8]. It has been postulated that the disproportionate focus on experiences with ICDs in the literature is owing to the perception that patients living with these devices experience greater treatment burdens and poorer health [9]. This focus is problematic as it obscures the experiences of the vast majority of patients living with a CIED, who generally receive a PPM [10].

The limited qualitative evidence suggests that other CIEDs present several substantial challenges for patients. Living with a PPM may lead to several lifestyle changes [11,12], issues of identity and body image for women [13], and disruptions in social participation and emotional state [14]. Despite this, many patients are able to adapt to living with their PPM [11-13], and some regain their desired way of life [14]. Furthermore, patients living with a S-ICD may experience a similar range of challenges as well as shock-related anxiety [15,16].

A patient’s knowledge about their CIED may be particularly important for overcoming these challenges and adapting to their device [17]. Systematic reviews suggest that patients living with an ICD feel that they lack sufficient knowledge about their device, which may be detrimental to their health management [5,6]. Similar concerns regarding knowledge deficits have been observed in people living with PPMs [18] and S-ICDs [15]. When faced with knowledge deficits, patients are increasingly turning to social media to gain insight into their conditions and to seek advice from their peers [19,20].

### Objectives

To date, limited research has assessed how patients living with a CIED interact with social media. There is evidence to suggest the utility of web-based peer support for patients living with a CIED, as studies indicate that patients living with an ICD desire lived experience groups to address knowledge deficits [21-23]. Web-based forums and social media for people with ICDs may allow for the provision of information and peer support [24,25], and this information may decrease experiences of shock-related anxiety [26]. However, social media information about ICDs can lack quality and overestimate the risks associated with living with these devices [27]. To understand the experiences of people living with a CIED and their interactions with social media, this study aimed to characterize the experiences and questions that patients posted to CIED-related communities hosted on the social media website Reddit.

## Methods

### Reddit as a Data Source

Reddit is a popular web-based social media platform that allows users who have created an account to submit, share, and discuss content. All content submitted to Reddit must be categorized into designated *subreddits*, each serving as a user-curated and managed community centered around distinct themes, interests, or subjects. The curated nature of these communities has been of particular interest to researchers, as subreddits have been used as a valuable resource for in-depth investigations into specific topics [28]. Reddit was selected as the focus of this study because data collection could be constrained to subreddits intended for the discussion of experiences with a CIED. Other social media platforms either lack this degree of curation or resort to private communities for discussing such topics. Accessing data from these private groups can raise ethical issues related to consent.

### Ethical Considerations

Participants in this study were considered to be Reddit users who had posted to subreddits related to CIEDs. This study received low-risk research ethics approval from the University of Adelaide School of Psychology Human Research Ethics Sub-Committee (22/27). As publicly available posts made to web-based communities can be considered textual documents [29-31], consent was not required from participants. However, unique ethical challenges are associated with the collection of web-based data [29,32]. Thus, ethics approval was granted on the grounds that all posts were publicly available, no contact was made with participants, and participants remained anonymous. Consequently, subreddits with specific rules prohibiting the collection of user data were excluded from this study, personal information was removed from the reported extracts, and each participant’s username was converted into a numeric pseudonym.

### Subreddit Selection and Data Collection

Reddit’s internal search engine was used to systematically identify suitable subreddits, and only those intended for people living with a CIED were included in the analysis (Multimedia Appendix 1). One subreddit intended for people with a CIED to discuss their experiences, questions, and concerns was identified and included in this study. At the time of data collection (April 17, 2022), the subreddit consisted of >1100 members, 484 top-level posts, and 4036 comments. Discussions of experiences related to living with a CIED may have occurred on subreddits related to more general medical issues. However, observation and searching of posts in these communities indicated that discussions on such topics were rare, making it difficult to include these posts given the data collection method used.

Consistent with Reddit’s terms of service, the website’s application programming interface was used to collect all top-level posts made to the subreddit [28]. Top-level posts, also known as initial posts, are the messages created by users to initiate a discussion thread on a specific topic within a subreddit. Only top-level posts were collected to develop an overview of the experiences shared by participants and how they engaged with the subreddit. Upon being granted access to the application programming interface, a script was created using Python (version 3.9.7) and the Python Reddit Application Programming Interface Wrapper (version 7.5.0) [33], which could systematically collect every top-level post made to the subreddit in chronological order (Multimedia Appendix 2). The script collected the title of each post, content, time of posting, and poster username.

After posts were collected using the Python script, they were each individually read to facilitate the exclusion of irrelevant posts. Only posts made by people who clearly indicated that they were currently living with a CIED were included in the analysis. Posts from people awaiting CIED implantation or caregivers were not included and will be used in subsequent analyses that aim to specifically represent their experiences. During the exclusion process, a register of participant demographic information was created based on their explicitly declared age, gender, type of CIED, and time lived with a CIED declared in each post. We considered each Reddit username as representing a distinct participant.

### Data Analysis

Conventional qualitative content analysis [34] was used to systematically classify the textual data from the collected posts into codes, subcategories, and categories. This was done to create a hierarchical framework that described the questions and experiences of participants. The analysis was inductive in nature and guided by the following research question: “What questions, and information about their experiences, do people living with a CIED post to communities intended for them on the social media website Reddit?” Each stage of the analysis was conducted using NVivo 12 Plus software (version 12.6.1.970; Lumivero), which allows researchers to organize and code qualitative data to identify meaningful insights. Captions for each code, subcategory, and category were determined by the authors based on the meaning and data contained within each analysis unit. As part of the conventional qualitative content analysis process, frequencies of occurrence were calculated based on how many participants produced textual data to which each unit of analysis applied. As participants could not be contacted for feedback, to enhance the credibility of the study, both the second and third authors (experienced health psychologists) evaluated the analysis. Typographical errors were corrected in all reported quotations.

## Results

### Data Collection

Of the 484 posts collected using the script, 186 were excluded. The excluded posts included 118 made by participants who did not have a CIED, 42 that were not usable in the analysis (eg, spam, links, or memes without context), and 26 made by participants who did not specify if they had a CIED. After exclusion, 298 posts that were made by 196 participants were included. The posting dates of the included posts ranged from January 24, 2018, to April 17, 2022 (the data collection date).

### Demographic Information

Table 1 illustrates the reported demographic characteristics of participants and their current CIED.

**Table 1:** Demographic characteristics reported by participants living with a cardiac implantable electronic device (CIED; N=196).

Characteristic			Value
**Gender, n (%)**			
	Men	26 (13.3)	
	Women	19 (9.7)	
	Not reported	151 (77)	
**Age (years)**			
	Values, median (range)	33 (22.0-39.5; 17-72)	
	Not reported, n (%)	117 (59.7)	
**Time lived with CIEDs (years), n (%)**			
	<1	45 (23)	
	1-5	16 (8.2)	
	5-10	9 (4.6)	
	10-15	6 (3.1)	
	15-20	8 (4.1)	
	20-25	3 (1.5)	
	25-30	3 (1.5)	
	30-35	3 (1.5)	
	Not reported	103 (52.6)	
**Current CIED type, n (%)**			
	PPM^a^	101 (51.5)	
	ICD^b^	61 (31.1)	
	S-ICD^c^	9 (4.6)	
	CRT^d^ device	6 (3.1)	
	Leadless PPM	4 (2)	
	Not reported	15 (7.7)	

^a^PPM: permanent pacemaker.

^b^ICD: implantable cardioverter defibrillator.

^c^S-ICD: subcutaneous implantable cardioverter defibrillator.

^d^CRT: cardiac resynchronization therapy.

### Analysis Structure: Categories, Subcategories, and Codes

**Figure 1:**
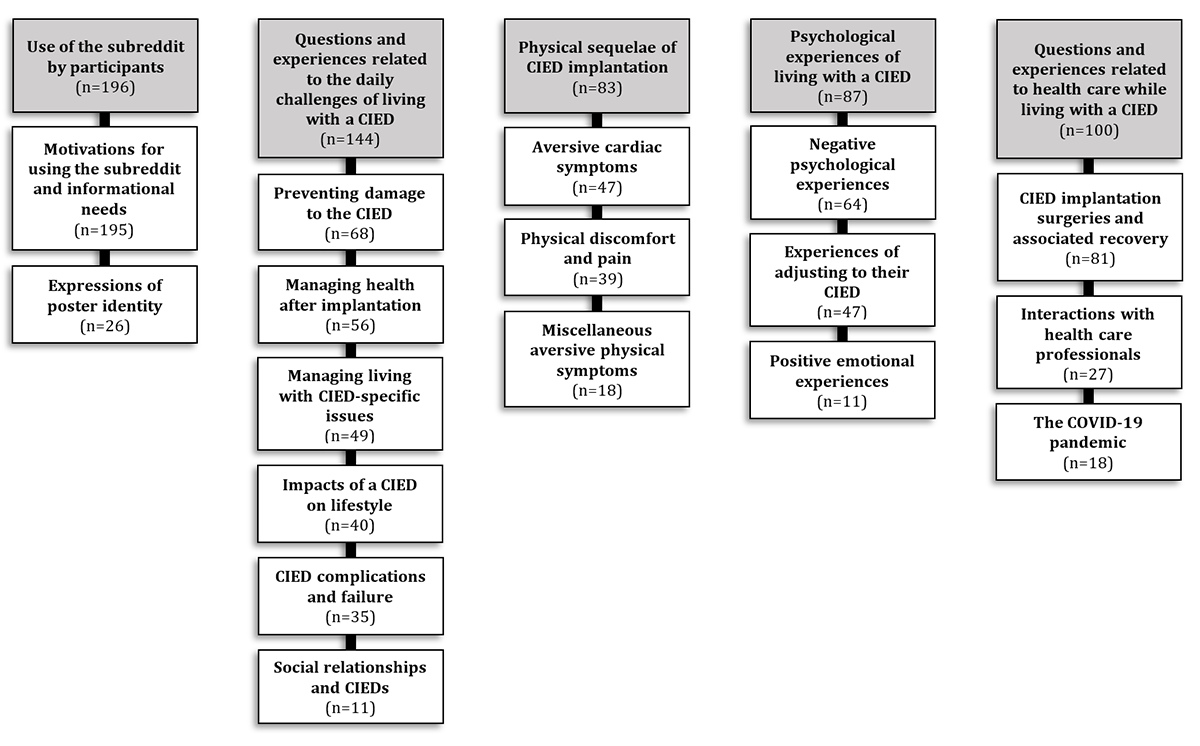
The experiences and questions of participants (N=196) with cardiac implantable electronic devices (CIEDs). Overarching categories (colored grey) and accompanying subcategories with occurrence frequencies.

Posts made to the subreddit were classified into 104 codes that were condensed into 17 subcategories and 5 overarching categories (refer to Multimedia Appendix 3 for the full analysis structure). The overarching categories and associated subcategories, with frequencies of occurrence for each, are shown in Figure 1.

#### Use of the Subreddit by Participants

The first category captured how participants engaged with the subreddit. The most frequent subcategory captured the participants’ declared motivations for posting and their associated informational needs. Most participants sought experiential knowledge of living with a CIED, followed by advice for dealing with their problems and answers to technical questions. The inability to find information about CIEDs, such as potentially sensitive topics or how to best live with these devices, also motivated participants to post:

I got a leadless pacemaker about a month ago. I am still confused about what I can do or not. Anyone else have one? I haven’t found much information on them either.

Participant 171

In addition to fulfilling their informational needs, the participants sought affirmation by asking the subreddit whether their experiences were normal and posted to thank the community for the help they had received. Participants were also motivated to post by an often explicitly stated desire to help others by sharing their experiences of living with a CIED:

I know there are a lot of emotions that go along with being different than “what’s normal,” and I would be more than happy to share my experiences, the challenges I’ve overcome and my philosophy on what it means to have such an amazing device.

Participant 21

The second subcategory captured how the participants expressed their identities on the subreddit. Younger participants specifically described themselves as such, and others described themselves as cyborgs or as belonging to a special club:

Have y’all ever realized that we’re basically cyborgs since our hearts are run by a piece of technology?

Participant 141

#### Questions and Experiences Related to the Daily Challenges of Living With a CIED

The second category captured discussions and questions related to the intricacies and daily challenges faced while living with a CIED. The most frequent subcategory captured participants’ experiences of, and questions about, preventing damage to their CIED. Concerns about electromagnetic interference, especially from emergent consumer technologies such as smartwatches, were most common. Participants discussed the restrictions placed on their physical activities to prevent damage to their CIED, such as discontinuing contact sports, and different methods of protecting their device from harm:

Were you able to continue the sports you were doing before? I understand contact sports are a no-no to avoid any blow to the ICD (but I’m guessing with protection it should be fine?)

Participant 88

Concerns about damage also stemmed from the use of recreational or prescription drugs, and the participants expressed a lack of information regarding these topics:

I know illegal substances are bad for you and we shouldn’t take them. But I still just want the information and can’t find any online. What would happen to my heart if I did a single line of cocaine? Or if I took molly once? Like, would I drop dead or get shocked? I searched online and couldn’t find any answers.

Participant 160

The second subcategory captured how participants evaluated and managed their health after implantation. Participants reported either improvements or declines in their health status. Most posts were related to exercise, and participants generally asked questions about the types of activities that were possible with a CIED. In addition, the participants reported their experiences with deliberate weight loss and associated pain-related complications owing to CIED protrusion:

One thing I’m noticing is that as I’ve started to slim down, my ICD is causing me more and more discomfort. Not via shocks or anything, just by kinda poking out. I can feel pressure on it whenever I lie down, whenever I sit in a chair or in the car, and it’s uncomfortable for a while, then gradually starts to become painful.

Participant 156

The third subcategory captured discussions related to the management of issues specific to being implanted with a CIED. Participants discussed ICD shocks, rubbing from external objects (eg, bra straps or seatbelts), CIED battery life, automated CIED checks, discomfort caused by CIED warning alarms, and medical alert identification information. Changes in CIED settings were reported to help alleviate some of the problems experienced by participants. Participants most frequently discussed the monitoring of device functioning using health tracking applications, CIED remote monitoring services, and consumer heart rate monitors. These efforts were often reported as a source of confusion:

I have 2 oximeters. I use them to take my pulse. Sometimes when I feel bad (with a lack of air feeling), I take my pulse. It has been in the 40s or 140s (too low or too high) sometimes during those episodes.... I’ve told my dr but he says that everything is ok because my PPM isn’t recording those events.... Is the oximeter accurate enough for us? Should I trust more the PPM than the oximeter?

Participant 86

The fourth subcategory captured the actual and potential impacts of having a CIED on the lifestyles of the participants. This subcategory included a diverse range of reciprocal impacts between having a CIED and work, driving, sleep, simple activities, school, sex and masturbation, diet, and pregnancy. The reported impacts of living with a CIED could be caused by its direct physical presence or the limitations of current technology:

I am a marine engineer and electrical engineer, currently looking for a work. I can no longer work with magnets or transformer stations, which has already led to me having to avoid certain job options. I’m scared of telling anyone outside my closest family, because employers might consider me damaged goods and reject me.

Participant 35

The fifth subcategory captured discussions related to the complications and failures of the participants’ CIEDs. The participants expressed concerns about lead dislocation or failure, device dislocation, and CIED malfunction. In addition, the participants reported their experiences of lead failure and CIED-related infections. Lead failures were perceived as impactful and were more commonly reported by younger and physically active participants:

Once I got to high school I ripped or broke my lead and feel like I have been accidentally doing this over the years because of my lead placement over my collar bone...I used to be very active so I have limited my activity and no longer do things like weightlifting. I am extremely careful and always conscious of my PPM.

Participant 168

The final subcategory captured the described perceptions of others toward CIEDs and the positive impacts of social support. Two participants raised concerns about the insensitive and negative perceptions of others toward their CIED:

I was out for dinner with my OH [other half] and 2 old friends and he drew attention to my PPM wound. They were all laughing and saying it looked like a mouth and that they could animate it and make it look funny.... They didn’t mean it maliciously, but it was really crushing and has massively knocked my confidence, just posting here because I’m struggling to find anyone who understands.

Participant 163

#### Physical Sequelae of CIED Implantation

The third category encompassed the physical consequences of having a CIED. The first subcategory captured the discussion of aversive CIED-related cardiac symptoms. This included the discussion of aversive heart rates, palpitations, shortness of breath, feelings of lightheadedness or fainting, loss of consciousness, feelings of dizziness, and fluid retention. Generally, participants sought the experiences of others and guidance on whether to seek medical care:

All of a sudden these past few days I am feeling dizzy all the time. Getting out of bed, laying in bed, outside, etc; has anyone else experienced anything similar to me? Should I contact my doctor? I get slightly lightheaded and my head starts to space out.

Participant 179

The second subcategory captured codes related to the discussion of the different experiences of pain and discomfort. Pain and discomfort were related to the direct physical presence of the CIED generator or leads, the surgical site, and the heart or chest. Participants also reported general experiences of bodily pain; these combined sensations were often complex and overlapping:

This stabbing/poking sensation in my heart has been present every moment of every day since my PPM was put in. Some positions hurt worse than others, such as leaning on your shoulder in bed at 3 am. The pain radiates up my jaw and into my molar. It’s driving me literally crazy.

Participant 12

The last subcategory captured miscellaneous aversive physical experiences that were not directly pain or cardiac related. The participants reported changes in body temperature, physical tiredness, bruising, feeling weak, twitching sensations, restlessness, excessive sighing or yawning, stomach problems, tingling sensations, and feeling shaky. Participants often inquired as to whether these symptoms had been experienced by other people and whether they should be concerned:

I recently got a PPM (a couple of months ago), and I’ve noticed my chest on the left side “twitches” or kind moves every time my heart beats. Has anyone else experienced this?

Participant 9

#### Psychological Experiences of Living With a CIED

The fourth category was related to the emotional and psychological aspects of living with and adapting to a CIED. The first subcategory captured the aversive psychological aspects of living with a CIED. Participants reported being worried, fearful or scared, anxious, depressed or sad, frustrated, traumatized or experiencing posttraumatic stress disorder, stressed, emotionally tired, feeling lost or empty, and cognitively impaired from their experiences. In addition, participants with an ICD reported anxiety related to potential shocks from their device. Experiences of worry and anxiety from having a CIED were often compounded by other aspects of their lives:

I’m 21, and I just got mine inserted yesterday.... I’ve been more anxious about almost everything and it doesn’t help that it’s my last semester of college so I’m also worrying about school work on top of trying to recover. I’ve had two different anxiety attacks just today.

Participant 38

The second subcategory captured how participants adapted to the challenges of living with a CIED. Participants discussed experiences with, and asked questions about, ways of coping with a CIED, returning to a normal lifestyle after implantation, trying to rebuild their confidence, and learning to trust their CIED and body. Participants expressed that their lives had changed after implantation, and 1 participant stated that they had become emotionally attached to their CIED. Not all participants were able to adapt to their CIED, and some participants even wanted theirs removed or deactivated. Similarly, some participants expressed hatred and resentment toward their CIED:

Need a battery change, haven’t made the appointment. Why? I hate this and I’m miserable. I’m afraid of it, it doesn’t make me feel safer, it’s taken my dreams of military service and flying away from me, I can’t do cool hobbies I’m interested in. When I bring this up to people they don’t get it. They think I should be thankful and appreciate that it might save my life. I don’t care. A longer life feels pointless when I don’t get to be happy.

Participant 5

Moreover, younger and more active participants expressed difficulties with accepting their CIED, which were unique to their age and health status:

I read the other posts and I guess I’m struggling with the whole “why me” crap. I live a healthy lifestyle, never smoked, and am in good health (besides having a battery in my chest). Like a lot of the other posts have stated, it’s more of a mind fuck than anything.

Participant 97

The least frequent subcategory captured positive emotional experiences related to living with a CIED. Participants reported being happy with or excited for having a CIED, proud of some aspect of living with a CIED, or grateful for being implanted with a CIED. Participants most often expressed positive emotions when their CIED presented clear benefits over undesirable alternatives:

Got the all clear to send back the LifeVest to Zoll [a wearable external defibrillator] after the longest 5 months of my life.... Cheers to life beyond the vest, and freedom from the electrodes, garments, and all the cords! I am most excited about my re-expanded wardrobe options and not lugging that box around my waist anymore.

Participant 115

#### Questions and Experiences Related to Health Care While Living With a CIED

The final category captured the experiences of, and questions about, health care after CIED implantation. The most frequent subcategory consisted of questions and experiences related to CIED surgeries and associated recovery. Topics included CIED replacement, implantation surgery, lead replacement and removal, implantation complications, additional surgeries to support heart function, costs associated with CIED surgeries, and bandages. Although many participants who discussed recovering from implantation surgery only described their experiences, those scheduled for a CIED replacement often expressed apprehension:

I am now scheduled to see an electrophysiologist this Thursday and I’m just.... Shocked. And really scared. I don’t know what to expect or how technology has changed.... I guess I just need to vent to people who understand but would also like some advice on recovery times, restrictions etc. Are PPMs like they were 17 years ago?

Participant 129

The second subcategory captured the interactions of participants with health care professionals. Participants discussed the medical advice they had received and their appreciation for some of the professionals with whom they had interacted, and 1 female participant expressed discomfort discussing sexual activity with her male physician. Participants most frequently expressed a sense of perceived ambivalence held by their health care professionals, and some participants disagreed with them about their symptoms:

The doctor says nothing is wrong with it and I shouldn’t be feeling it [pulsating heart pain and aversive cardiac symptoms] but yet I still do. I’m not getting any answers or help from the cardiologist, so I just don’t know what to think anymore.

Participant 169

The third subcategory captured the impact of the COVID-19 pandemic on the experiences of participants, such as public health restrictions limiting health care opportunities. In addition, participants reported possible complications from the COVID-19 vaccine and raised questions about who can access it or concerns about potential side effects:

Has anyone had any discussions with their cardiologist or GP regarding receiving Pfizer of Astra Zeneca vaccinations for COVID-19? I have complete heart block, and I have some preliminary concerns about receiving the vaccination in the fear that it may not have been adequately tested.

Participant 154

## Discussion

### Principal Findings and Comparison With Prior Work

To the best of our knowledge, this is the first study to explore how people living with a CIED use the social media website Reddit. One subreddit analyzed was used by participants living with a CIED to find information, share a range of experiences, and render support to their peers through the provision of knowledge and affirmation. A number of insights are presented, including how participants engaged with the subreddit, their experiences of living with a CIED, the impacts of physical activity and age on their experiences, and their experiences during the COVID-19 pandemic.

The ability to receive and share information formed the central way users engaged with the subreddit. Participants with different types of CIED posted to the subreddit to receive experiential knowledge, advice to overcome the challenges they faced, fill unmet educational needs, and have their experiences affirmed by others living with a CIED. Similar patterns of information seeking have been observed in people living with an ICD [25]. In addition, CIED ownership was demarcated as a unifying group identity, and participants expressed a desire to provide support to others living with these devices. Taken together, these patterns of behavior are consistent with theoretical conceptualizations of peer support [35,36] and build on research that has explored the natural occurrence of these phenomena in social media contexts [37,38].

The experiences described by participants who were predominately implanted with a PPM largely concurred with previous qualitative studies that have explored the challenges faced by people living with ICDs [5,6], who represent one of the most studied CIED patient groups [9]. The findings were also consistent with the previously reported experiences of patients living with PPMs [11-14] and S-ICDs [15]. As previous studies have almost exclusively used interview-based methodologies with in-hospital and community populations, this suggests that the concerns expressed by people with a CIED may be comparable between web-based platforms and face-to-face investigations. This said, social media serves as a way for people living with a CIED to ask questions about sensitive topics that they may not feel comfortable discussing in formalized research contexts. The finding that the subreddit was used to discuss drug use was a novel contribution to the existing literature, underscoring the need for enhanced education in this specific domain.

Compared with previous literature and available demographic data, participants in this study appeared to be younger and more physically active than the average person living with a CIED [10]. In the current literature, this population represents a novel demographic that may be linked to the web-based nature of Reddit, which necessitates a higher level of technological literacy for engagement. As an indication of the unique demographics present in the sample, no discussion regarding the end-of-life discontinuation of device treatments was observed, although this is a common topic in the current CIED literature [8]. This was most likely owing to the younger age and greater physical activity levels observed across the data. In addition, individuals with a PPM, the most prevalent subgroup in our sample, may have had a more favorable prognosis compared with other device populations. Interestingly, the discussion of end-of-life considerations was observed in posts from caregivers who were excluded from the analysis. We plan to conduct subsequent analyses of the posts made by caregivers to provide comprehensive insight into their experiences.

The reduced age and increased activity level of participants provide a number of novel insights into the experiences of patients living with a CIED. Similar to previous studies of people living with an ICD, restrictions placed on participation in physical activities could be substantially impactful for the experiences of participants with any CIED [6,21,22,39]. To overcome these restrictions, participants discussed a range of efforts to protect their CIED from damage. This included prevention efforts, such as limiting physical activity and avoiding danger, and protection efforts through the use of sports guards. Protection also involved participant comfort, with cushioned pads reported as being used to assist with external objects rubbing against the device; this included seatbelts [40] and bra straps [16], which have been previously reported as sources of discomfort. Furthermore, participants often saw their CIED as at risk of failure and deemed it necessary to monitor the functioning of their device through various domestically available technologies. We are unaware of previous studies that have evaluated these topics.

Despite these activity restrictions and associated difficulties, the participants viewed the continuation of exercise to stay healthy as important. Participants did not discuss CIED-related body image concerns, which is a common topic in the literature [13,16,41-43]. Instead, body image concerns focused on deliberate postimplantation weight loss attempts, often in the context of exercise. Participants attempting to lose weight reported pain and discomfort caused by the thinning of tissue around their implantation site, and the medical literature has previously drawn associations between decreases in BMI and this phenomenon [44]. We are unaware of previous studies evaluating the experiences of deliberate weight loss while living with a CIED.

The experiences of individuals living with a CIED during the COVID-19 pandemic emerged as a notable topic of discussion on the subreddit. This topic has received little attention in the current literature. Delays in accessing health care and the difficulties created by public health approaches used to manage the pandemic were discussed. In addition, there is a need for targeted pandemic-related information for people living with a CIED, as questions were posted about the availability of vaccines and possible complications from vaccination.

### Limitations

The results should be interpreted within the context of the limitations posed by the methodology and sample used. Proferes et al [28] asked whether Reddit data only provides insight into phenomena specific to the characteristics of the website. The findings of this study may reflect the specific qualities of the subreddit analyzed, as it could have been the last resort for people unable to receive adequate health services. Reddit users also tend to be younger than the general population [45], and social media data may overrepresent people of higher socioeconomic status [46]. Furthermore, participants could not be contacted to clarify their postings, and they may have inaccurately recounted their experiences [47]. Although this may be the case, this study used a novel systematic approach for the collection of naturalistic data, which allowed deep insight into the experiences and needs of the participants.

### Implications

The findings present several implications for the improvement of care and outcomes for patients living with a CIED. Participants living with a CIED experienced numerous biological, psychological, and social challenges associated with their device; thus, an understanding of postimplantation quality of life should consider holistic perspectives. In doing so, health care professionals and researchers must develop appropriate patient resources and supports that address the diversity of patient experiences, which could include the use of educational interviews tailored to the lifestyles of patients [48]. As a majority of participants desired experiential knowledge, the creation of formalized peer support networks should also be explored, as they have demonstrated benefits for people living with chronic health conditions [49,50]. Further work is needed to explore the nuances and differences in patient experiences between devices.

### Conclusions

This study analyzed posts from a community on the social media website Reddit intended for people living with a CIED to characterize the informational needs and experiences of patients. In this context, participants expressed a desire for a range of information, with the majority requesting experiential information. Participants living with a CIED experienced a range of challenges, including the need to adapt to living with a device, difficulties with navigating health care, psychological difficulties, and various aversive physical sequelae. These challenges were often most deeply impactful for younger and more physically active participants. This study demonstrated that social media represents a way in which people living with a CIED may engage in peer support to help address the diverse and unique challenges that they may face. Health care professionals may wish to draw on peer support and other forms of aid to help their patients overcome the challenges of living with these devices.

## Appendix

Multimedia Appendix 1 Systematic search terms and exclusion criteria.

Multimedia Appendix 2 Data collection Python script using the Python Reddit Application Programming Interface Wrapper.

Multimedia Appendix 3 Structure of the categories, subcategories, and associated codes, with occurrence frequencies calculated for the 196 participants living with a cardiac implantable electronic device.

## Abbreviations

CIED: cardiac implantable electronic device
CRT: cardiac resynchronization therapy
ICD: implantable cardioverter defibrillator
PPM: permanent pacemaker
S-ICD: subcutaneous implantable cardioverter defibrillator

## Data Availability

The data collected and analyzed during this study are available from the corresponding author upon reasonable request.
