# Supplementary material for: The Information Needs and Experiences of People Living With Cardiac Implantable Electronic Devices: Qualitative Content Analysis of Reddit Posts
Source: JMIR Cardio. 2023 Nov 1;7:e46296. doi: 10.2196/46296 (PMC10652197; doi:10.2196/46296)
Supplement: Multimedia Appendix 1 [file cardio_v7i1e46296_app1.doc]

**Multimedia Appendix 1**

## Systematic Search Terms and Exclusion Criteria

### Search Terms

The following search terms were used individually: “artificial pacemaker,” “cardiac implantable electronic device,” “cardiac pacemaker,” “pacemaker,” “permanent pacemaker,” “ICD,” “CRT-D,” “cardiac resynchronization therapy,” and “implantable cardioverter defibrillator.”

### Search Settings

Set to search for “subreddits” only

### Exclusion Criteria

Subreddits would be excluded for the analysis based on four criteria:

- Subreddits not intended for people with CIEDs.
- Subreddits that have not been active for the past 6 months.
- Subreddits intended for the general discussion of medical problems that are not specific to people with CIEDs.
- The target audience of the subreddit is unclear.
