# Supplementary material for: The Information Needs and Experiences of People Living With Cardiac Implantable Electronic Devices: Qualitative Content Analysis of Reddit Posts
Source: JMIR Cardio. 2023 Nov 1;7:e46296. doi: 10.2196/46296 (PMC10652197; doi:10.2196/46296)
Supplement: Multimedia Appendix 2 [file cardio_v7i1e46296_app2.doc]

**Multimedia Appendix 2**

Data collection python script using the Python Reddit Application Programming Interface Wrapper (PRAW).

from concurrent.futures import thread

import praw

import pandas as pd

from praw.models import MoreComments

from sympy import true

reddit = praw.Reddit(client_id='******************', client_secret='*****************',

user_agent="Comment Extraction (by /u/**********)", timeout = 60)

a=0

posts = []

ml_subreddit = reddit.subreddit('XXXXXX')

for post in ml_subreddit.new(limit=999):

posts.append([post.title, post.author, post.selftext, post.created_utc])

a+=1

posts.append(["~~~~~~~", "~~~~~~~", "~~~~~~~","~~~~~~~"])

posts = pd.DataFrame(posts, columns=['title_of_parent', 'author', 'content', 'date_created'])

print("posts num:", a)

posts.to_csv('data_fin.csv', encoding='utf-8', index=False)
