# Supplementary material for: The Information Needs and Experiences of People Living With Cardiac Implantable Electronic Devices: Qualitative Content Analysis of Reddit Posts
Source: JMIR Cardio. 2023 Nov 1;7:e46296. doi: 10.2196/46296 (PMC10652197; doi:10.2196/46296)
Supplement: Multimedia Appendix 3 [file cardio_v7i1e46296_app3.doc]

**Multimedia Appendix 3**

Structure of Categories, subcategories, and associated codes, with occurrence frequencies calculated for all 196 participants living with a Cardiac Implantable Electronic Device (CIED).

| Category | Subcategory | Codes | *n* | % of *N* |
| --- | --- | --- | --- | --- |
| Use of the Subreddit by Participants | Motivations for Using the Subreddit and Informational Needs | Seeking the experience of others | 122 | 62.2% |
|  |  | Seeking advice from others | 80 | 40.8% |
|  |  | Sharing firsthand experiences of living with a CIED | 56 | 28.6% |
|  |  | Seeking answers to technical questions | 18 | 9.2% |
|  |  | Giving thanks to the community | 16 | 8.2% |
|  |  | Seeking information not found elsewhere | 14 | 7.1% |
|  |  | Asking whether their experiences are normal | 12 | 6.1% |
|  | Expressions of Poster Identity | Identifying as a younger person | 12 | 6.1% |
|  |  | Identifying as a member of a “club” | 8 | 4.1% |
|  |  | Identifying as a “cyborg” | 8 | 4.1% |
| Questions and Experiences Related to Living With a CIED | Preventing Damage to the CIED | Concerns about electromagnetic interference | 32 | 16.3% |
|  |  | Restrictions placed on activity to prevent damage to the CIED | 30 | 15.3% |
|  |  | Methods of protecting the CIED from damage | 10 | 5.1% |
|  |  | Concerns about recreational drug use | 5 | 2.6% |
|  |  | Concerns about hormonal drug use | 1 | 0.5% |
|  | Managing Health After Implantation | Questions about and experiences of exercising | 35 | 17.9% |
|  |  | Perceived improvements in health status | 16 | 8.2% |
|  |  | Perceived declines in health status | 13 | 6.6% |
|  |  | Experiences with deliberate weight loss | 4 | 2.0% |
|  | Managing Living With CIED-Specific Issues | Monitoring their CIED and heart function | 21 | 10.7% |
|  |  | Experiences of and questions about ICD shocks | 15 | 7.7% |
|  |  | Experiences of having their CIED settings changed | 9 | 4.6% |
|  |  | External objects rubbing against the CIED | 9 | 4.6% |
|  |  | CIED Battery life | 5 | 2.6% |
|  |  | Concerns about automated CIED checks | 4 | 2.0% |
|  |  | Discomfort from CIED warning alarms | 3 | 1.5% |
|  |  | Questions about medical alert identification | 2 | 1.0% |
|  | Impacts of a CIED on Lifestyle | Impacts on work and career aspirations | 12 | 6.1% |
|  |  | Impacts on driving | 11 | 5.6% |
|  |  | Impacts on sleep | 11 | 5.6% |
|  |  | Impacts on performing simple activities | 7 | 3.6% |
|  |  | Impacts on school | 3 | 1.5% |
|  |  | Impacts on sex and masturbation | 2 | 1.0% |
|  |  | Impacts on diet | 1 | 0.5% |
|  |  | Impacts on pregnancy and child birth | 1 | 0.5% |
|  | CIED Complications and Failure | Concerns about lead dislocation or failure | 13 | 6.6% |
|  |  | Experiences of CIED lead failures | 10 | 5.1% |
|  |  | Concerns about CIED dislocation | 8 | 4.1% |
|  |  | Concerns about CIED malfunction | 4 | 2.0% |
|  |  | Experiences of CIED related infections | 4 | 2.0% |
|  | Social Relationships and CIEDs | General perceptions of others towards their CIED | 7 | 3.6% |
|  |  | The positive impacts of social support | 3 | 1.5% |
|  |  | Insensitive and negative perceptions of others | 2 | 1.0% |
| Questions and Experiences Related to Health Care While Living With a CIED | CIED Implantation Surgeries and Associated Recovery | Recovery from implantation surgery | 46 | 23.5% |
|  |  | CIED replacement | 28 | 14.3% |
|  |  | General Implantation surgery discussions | 20 | 10.2% |
|  |  | Lead replacement and removal | 17 | 8.7% |
|  |  | Implantation complications | 7 | 3.6% |
|  |  | Additional surgeries to support heart function | 7 | 3.6% |
|  |  | Costs associated with CIED related surgeries | 4 | 2.0% |
|  |  | Surgical bandages | 1 | 0.5% |
|  | Interactions With Health Care Professionals | Perceived health care professional ambivalence | 14 | 7.1% |
|  |  | Medical advice from health care professionals | 13 | 6.6% |
|  |  | Disagreement about symptoms | 7 | 3.6% |
|  |  | Appreciation for health care professionals | 3 | 1.5% |
|  |  | Uncomfortable discussing with a male physician | 1 | 0.5% |
|  | The COVID-19 Pandemic | Impacts of the COVID-19 pandemic on experiences | 12 | 6.1% |
|  |  | COVID-19 vaccine questions and concerns | 5 | 2.6% |
|  |  | COVID-19 vaccine complications | 3 | 1.5% |
| Psychological Experiences of Living With a CIED | Negative Psychological Experiences | Feeling worried about living with a CIED | 30 | 15.3% |
|  |  | Feeling fearful or scared about living with a CIED | 19 | 9.7% |
|  |  | Anxious about living with a CIED | 14 | 7.1% |
|  |  | Depressed and sad about living with a CIED | 12 | 6.1% |
|  |  | Frustrated due to aspects of living with a CIED | 9 | 4.6% |
|  |  | Implantable cardioverter-defibrillator related shock anxiety | 7 | 3.6% |
|  |  | Traumatised or experiencing PTSD from having a CIED | 6 | 3.1% |
|  |  | Stressed about living with a CIED | 5 | 2.6% |
|  |  | Emotionally tired from living with a CIED | 3 | 1.5% |
|  |  | Feeling lost or empty due to their experiences | 3 | 1.5% |
|  |  | Experiencing cognitive impairment due to surgery | 2 | 1.0% |
|  | Experiences of Adjusting to Their CIED | Ways of coping with a CIED | 12 | 6.1% |
|  |  | Difficulty accepting due to age and health | 10 | 5.1% |
|  |  | Returning to a normal lifestyle after implantation | 10 | 5.1% |
|  |  | Difficulty adapting to living with a CIED | 6 | 3.1% |
|  |  | Trying to rebuild their confidence after implantation | 5 | 2.6% |
|  |  | Descriptions of their life being changed by the CIED | 4 | 2.0% |
|  |  | Feeling resentment or hatred towards their CIED | 4 | 2.0% |
|  |  | Wanting CIED removal or deactivation | 3 | 1.5% |
|  |  | Learning to trust the CIED and their body | 3 | 1.5% |
|  |  | Becoming emotionally attached to the CIED | 1 | 0.5% |
|  | Positive Emotional Experiences | Happy with or excited for having a CIED implanted | 5 | 2.6% |
|  |  | Proud to be living with a CIED | 4 | 2.0% |
|  |  | Gratitude for being implanted with a CIED | 2 | 1.0% |
| Physical Sequelae of CIED Implantation | Aversive Cardiac Symptoms | Aversive heart rates | 29 | 14.8% |
|  |  | Aversive heart palpitations | 11 | 5.6% |
|  |  | Shortness of breath | 10 | 5.1% |
|  |  | Feelings of light-headedness or fainting | 5 | 2.6% |
|  |  | Losses of consciousness | 4 | 2.0% |
|  |  | Feelings of dizziness | 3 | 1.5% |
|  |  | Fluid retention | 1 | 0.5% |
|  | Physical Discomfort and Pain | CIED related discomfort and pain | 16 | 8.2% |
|  |  | General discomfort and pain | 11 | 5.6% |
|  |  | Surgical site discomfort and pain | 11 | 5.6% |
|  |  | Heart or chest related discomfort and pain | 10 | 5.1% |
|  | Miscellaneous Aversive Physical Symptoms | Body temperature changes | 5 | 2.6% |
|  |  | Physically tired | 5 | 2.6% |
|  |  | Bruising | 3 | 1.5% |
|  |  | Feeling weak | 2 | 1.0% |
|  |  | Twitching sensations | 2 | 1.0% |
|  |  | Restlessness | 1 | 0.5% |
|  |  | Excessive sighing and yawning | 1 | 0.5% |
|  |  | Stomach problems | 1 | 0.5% |
|  |  | Tingling sensations | 1 | 0.5% |
|  |  | Feeling shaky | 1 | 0.5% |
